# Dexmedetomidine, an alpha 2A receptor agonist, triggers seizures unilaterally in GAERS during the pre-epileptic phase: does the onset of spike-and-wave discharges occur in a focal manner?

**DOI:** 10.3389/fneur.2023.1231736

**Published:** 2023-12-11

**Authors:** Melis Yavuz, Pelin İyiköşker, Nursima Mutlu, Serra Kiliçparlar, Öykü Hazal Şalci, Gökçen Dolu, Elif Nur Kaymakçilar, Serdar Akkol, Filiz Onat

**Affiliations:** ^1^Department of Pharmacology, Faculty of Pharmacy, Acibadem Mehmet Ali Aydinlar University, Istanbul, Türkiye; ^2^Faculty of Pharmacy, Acibadem Mehmet Ali Aydinlar University, Istanbul, Türkiye; ^3^Department of Biotechnology and Genetics, Institute of Science, Istanbul University, Istanbul, Türkiye; ^4^Department of Neurology, University of Alabama at Birmingham, Birmingham, AL, United States; ^5^Department of Medical Pharmacology, School of Medicine, Acibadem Mehmet Ali Aydinlar University, Istanbul, Türkiye; ^6^Institute of Neurosciences, Acibadem Mehmet Ali Aydinlar University, Istanbul, Türkiye

**Keywords:** GAERS, spike-and-wave discharges, unilateral seizures, α_2A_AR, pre-epileptic, dexmedetomidine, pups, epileptiform

## Abstract

**Introduction:**

The genetic absence epilepsy rat from Strasbourg (GAERS) is a rat model for infantile absence epilepsy with spike-and-wave discharges (SWDs). This study aimed to investigate the potential of alpha 2A agonism to induce seizures during the pre-epileptic period in GAERS rats.

**Methods:**

Stereotaxic surgery was performed on male pups and adult GAERS rats to implant recording electrodes in the frontoparietal cortices (right/left) under anesthesia (PN23–26). Following the recovery period, pup GAERS rats were subjected to electroencephalography (EEG) recordings for 2 h. Before the injections, pup epileptiform activity was examined using baseline EEG data. Dexmedetomidine was acutely administered at 0.6 mg/kg to pup GAERS rats 2–3 days after the surgery and once during the post-natal (PN) days 25–29. Epileptiform activities before injections triggered unilateral SWDs and induced sleep durations, and power spectral density was evaluated based on EEG traces.

**Results:**

The most prominent finding of this study is that unilateral SWD-like activities were induced in 47% of the animals with the intraperitoneal dexmedetomidine injection. The baseline EEGs of pup GAERS rats had no SWDs as expected since they are in the pre-epileptic period but showed low-amplitude non-rhythmic epileptiform activity. There was no difference in the duration of epileptiform activities between the basal EEG groups and DEX-injected unilateral SWD-like-exhibiting and non-SWD-like activities groups; however, the sleep duration of the unilateral SWD-like-exhibiting group was shorter. Power spectrum density (PSD) results revealed that the 1.75-Hz power in the left hemisphere peaks significantly higher than in the right.

**Discussion:**

As anticipated, pup GAERS rats in the pre-epileptic stage showed no SWDs. Nevertheless, they exhibited sporadic epileptiform activities. Specifically, dexmedetomidine induced SWD-like activities solely within the left hemisphere. These observations imply that absence seizures might originate unilaterally in the left cortex due to α_2A_AR agonism. Additional research is necessary to explore the precise cortical focal point of this activity.

## Introduction

Absence seizures are the most common type of primary generalized epileptic seizures, and they are distinguished by the presence of spike-and-wave discharges (SWDs) in the electroencephalogram (EEG). These discharges are believed to be caused by cortico-thalamocortical mechanisms. Genetic animal models have played a crucial role in elucidating the underlying causes of absence seizures (1).

Idiopathic, non-convulsive, and generalized absence seizures are the three types of seizures (2). The EEG shows bilaterally coordinated and symmetrical SWDs at 3 Hz during absence seizures (3). Gibbs et al. (4) found the association between behavioral unconsciousness and the presence of 3–4 Hz spike-and-slow wave complexes on the EEG in 1935. When depth electrodes were placed into the thalamus of a patient with absence epilepsy, bilateral and synchronous SWDs were seen (5).

In the domain of absence epilepsy research, two commonly used rat models for studying absence epilepsy are the rats of Strasbourg origin [genetic absence epilepsy rat from Strasbourg (GAERS)] and Rijswijk origin (WAG/Rij). The GAERS rats from Strasbourg are a useful model in which behavioral components accompany SWDs, similar to seizures observed in childhood absence epilepsy (6). Absence epileptic seizures do not appear immediately after birth in GAERS rats but emerge after a latent period. These seizures typically arise between 40 and 120 days, with a peak at ~60 days, when the first SWDs appear on the EEG, making GAERS rats an established model for studying absence epilepsy. As the rats aged, the frequency, and length of these discharges increased. We also observed in the EEGs previously that SWDs do not appear in GAERS rats until the 30th day after birth (7). This reflects that the pre-epileptic period, a silent phase of epileptogenesis, is anticipated to unfold (8), and our understanding of this crucial developmental stage is still limited.

The role of alpha 2A adrenergic receptors (α_2A_AR), a specific subtype of α_2_AR known to be involved in the generation and sustainability of SWDs, has been extensively investigated (9–12). In rats, a decrease in noradrenergic and dopaminergic activity has been shown to promote the occurrence of absence-like seizures (13). A previous study has shown that activating α_2A_AR receptors with the antagonist atipamezole efficiently decreased SWDs in adult GAERS rats (14) but activating α_2A_AR with agonist dexmedetomidine established a model of status epilepticus similar to prolonged absence seizures (15). In this study, dexmedetomidine also induced a state of switch from status to sleep and back from sleep to status (15). These findings help to show them as key players in the involvement of SWDs.

The generation of SWDs has long been debated, with two main theories emerging. Among these, the cortical theory has garnered a larger following. The somatosensory cortex has received much attention in this area and has been established as a key player in SWD generation through numerous studies (16, 17). Bancaud et al. (18) remarkable research on human patients provided direct evidence that initiating a focal discharge in the frontal cortex later propagates to the cortico-cortical pathways.

Further evidence of cortical involvement, particularly in the frontal and parietal regions, comes from EEG/fMRI data of patients with Rolandic epilepsy, where thalamic signals were found to follow cortical signals with higher amplitude (19). These findings are confirmed by neuropathological discoveries that confirm the cortical influence on SWDs. In addition, recent studies address that dexmedetomidine may facilitate seizure expression with peripheral somatosensory stimulation in rats, and interestingly, these seizures are focal initially (20, 21). These studies mention the high-frequency oscillations (ripples and fast ripples) preceded by the induction of these seizures. In this study, we aimed to investigate the SWDs before they were fully expressed in the GAERS model. Specifically, our objective is to determine whether α_2A_AR stimulation could induce SWD activity. We aimed to understand better the early stages of SWD and the potential role of α_2A_AR in SWD initiation.

## Methods

### Animals and experimental groups

The study was performed at Acibadem Mehmet Ali Aydinlar University Medical Experimental Application and Research Center (DEHAM) and was approved by the Acibadem University Experimental Animals Local Ethics Committee under decision number ACUHAYDEK2020/51.

The GAERS rats were bred and housed in a controlled environment in the animal care and production area. The room was set to a 12-h light/12-h dark cycle, and the temperature was 24 ± 2°C. The rats had unrestricted access to standard rat chow and drinking water. To preserve the GAERS strain's specific absence of epilepsy characteristics, inbreeding practices were used from the GAERS strain with 7- to 11-Hz spontaneous SWDs (1). The animals were housed in pairs in cages before the surgical procedures. However, after the completion of the stereotaxic surgeries, each cage accommodated only one animal to ensure proper post-surgery care and monitoring.

Male GAERS offspring rats (PN 23–26) obtained from Acibadem University DEHAM, still in their epileptogenesis period and weighed between 25 and 45 g, did not yet express SWDs. The animals were implanted with electrodes on the PN 23-26 and EEGs were performed on the PN 25–29. The offspring rats were connected to the EEG and their postnatal days were between PN23 and PN26, their EEGs were recorded once after 2–3 days after the surgery and once. The SWD expressions were confirmed as none by the 20-min baseline EEG. The waiting period after stereotaxic surgery was a minimum of 2 days.

### Stereotaxic surgery

Stereotaxic surgery was performed under ketamine/chlorobutanol (100 mg/kg, VetaKetam; Oruç Özel Vet. Hiz. Hay. Ve Gida San. Tic. LLC.) and xylazine (10 mg/kg; Rompun, 2%; Bayer HealthCare, LLC) anesthesia, both of which were administered intraperitoneally (*i.p*.) to all experimental group rats. The heads of the animals were first placed in the stereotaxy device after the ear bars were fixed in the anterior chamber of the stereotaxy device (Stoelting Model 51600, Stoelting Co., Illinois). Four stainless steel screws with insulated wires were implanted bilaterally to the right and left frontal bones over the cortex rat brain atlas (22) according to the coordinates provided by reference to the bregma point and adapted to the pup animals (right/left frontal; AP: +2.2, ML: ±1.5; parietal AP: −2.9, ML: ±1.5). The opposite ends of the cables, which had previously been attached to pins, were soldered to the tiny connections generated by cutting the male VGA connectors into four threads with multiwire conductor cables using phosphoric acid and a soldering device instrument. Dental acrylic was used to cover and secure the electrodes and cables to the skull. Following the surgery, a 0.9% isotonic sodium chloride solution was injected subcutaneously to supply any possible fluid loss in the animal.

### EEG recordings and analysis

After the electrodes were placed by stereotaxic surgery, the animals were allowed to rest for 2 days. The animals' basal activity was then recorded to analyze the epileptiform activities and freezing behaviors. The signals from the electrodes were transferred to ML 136 bioamplifier (ADInstruments) for EEG recording using the EEG recording wire. The amplifier signals were sent to the computer using the Powerlab system (PowerLab8S ADI Instruments, Oxfordshire, UK). The frequency filter is used in the 1- to 40-Hz band. EEG recordings on the computer were analyzed with the LabChart 8.0 program. Epileptiform activity analysis was performed by manually selecting the EEG activity occurring in both cortices of the animals between the basal EEG from groups, DEX injected-unilateral SWD-like-exhibiting and non-SWD-like activities groups over the duration of 1 sec to improve the accuracy of power spectral analysis (below) by increasing sampled length of time. Sleep time was also monitored during the EEG and video recordings.

### Dexmedetomidine injections

Following the recording of basal activity, 0.6 mg/kg dexmedetomidine was injected *i.p*. acutely into the animals. After the injection of dexmedetomidine, the EEG was recorded for two more hours, and a power spectrum density (PSD) analysis was performed.

### PSD analysis

The SWD and SWD-like EEG data were preprocessed using Fieldtrip (23) and custom MATLAB scripts (R2022a, MathWorks, Natick, Massachusetts). SWD activity was obtained and is shown as an example (24). Figure 2 shows the EEG data after bandpass filtering (with zero-phase, third-order, Butterworth filter using bandpass function in MATLAB, between 1 and 40 Hz). Multitaper spectral decomposition was used at 0.25 Hz frequency steps with discrete prolate spheroidal sequences and 1 Hz multitapers. PSD was the amplitude of the time-frequency decomposition calcuated for each frequency.

### Statistical analysis

All statistical analyses were performed with GraphPad Prism version 9.5.0 (GraphPad Software, San Diego, USA). Descriptive statistics were used to examine the total, mean duration, and number of SWD-like activities and the percentage of animals in which this activity was triggered (the unilateral SWD-like-exhibiting group). The unpaired Student's *t*-test was used to compare the mean duration of epileptiform activities and sleep (*p* < 0.05 and *p* < 0.01).

## Results

### Proportion of animals with unilateral SWDs and the total, mean duration, and number of SWD-like activities

**Figure 1:**
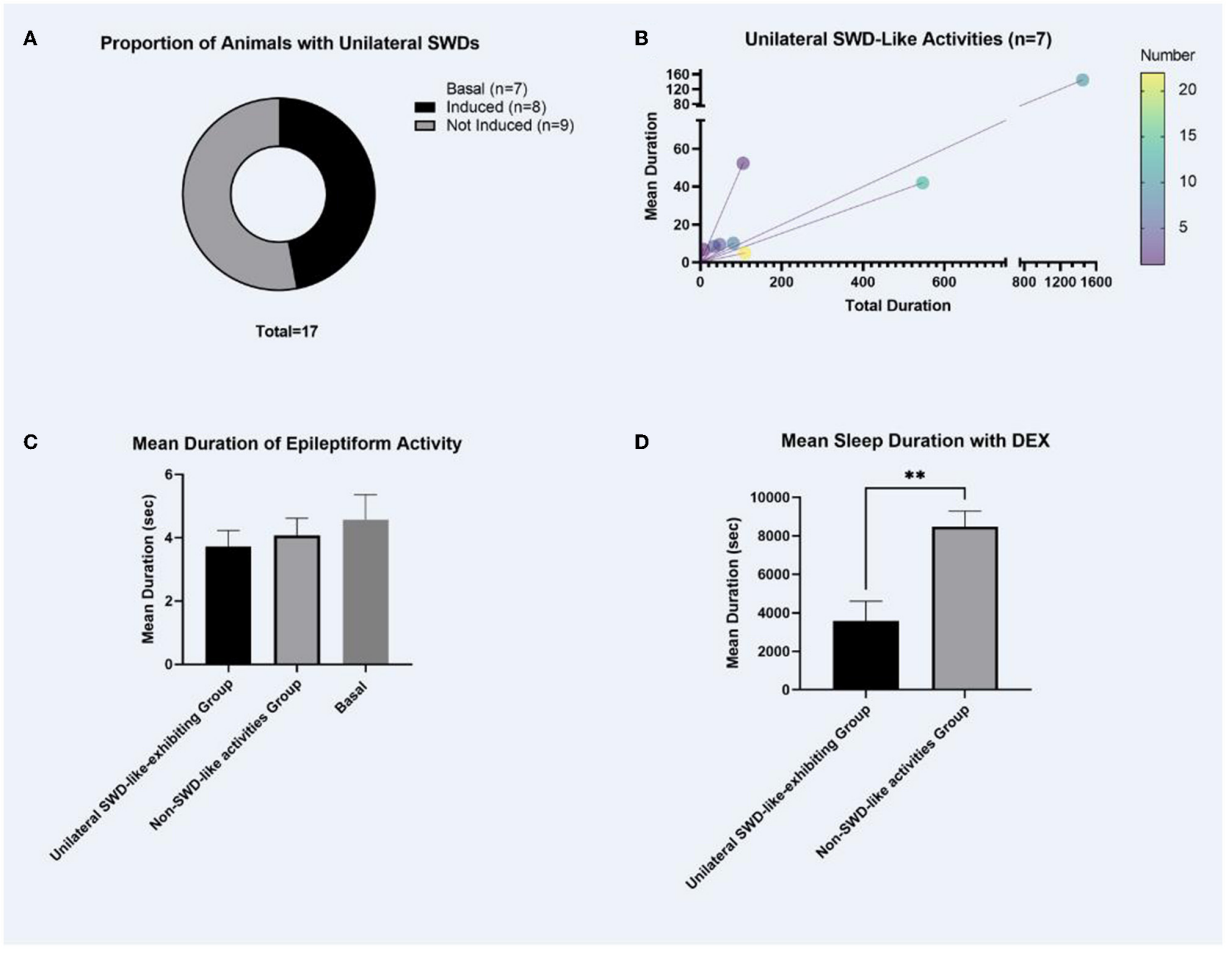
The proportion of animals with unilateral SWDs, the total mean duration of SWD-like activities, the mean duration of epileptiform activities, and the sleep duration following dexmedetomidine injections. **(A)** As expected, the baseline EEGs of pup GAERS rats did not show matured SWDs; it showed low amplitude non-rhythmic epileptiform activity. Mature SWDs occur after post-natal 30 in adult GAERS. Following a 0.6-mg/kg dexmedetomidine injection, unilateral SWD-like activity was observed in 47% (8/17) of the rats, as illustrated in **(A)**. **(B)** Characteristics of Unilateral SWD-like Activity. Unilateral SWD-like activities were analyzed within the group of animals exhibiting this response shown in **(A)** (*n* = 8). **(C)** Duration of Epileptiform Activities. Comparison of the duration of epileptiform activity between the “unilateral SWD-like-exhibiting group” and the “non-SWD-like activities group” revealed no significant difference in the **(C)**. **(D)** Sleep Duration Following Dexmedetomidine Injections. In the “unilateral SWD-like-exhibiting group,” the mean sleep duration was significantly shorter compared to the “non-SWD-like activities group.” No sleep activity was observed on the baseline EEG data as expected. The data were given as mean ± *SEM*. **p < 0.01 (significant difference).

The baseline EEGs of pup GAERS rats did not show SWD as expected but showed low-amplitude non-rhythmic epileptiform activity. A 0.6-mg/kg dexmedetomidine injection generated unilateral SWD-like activity in 47% of the rats (Figure 1A).

The descriptive statistics of unilateral SWD-like activities in the left cortex of animals that were triggered were analyzed (the unilateral SWD-like-exhibiting group). All SWD-like activities between the animals (*n* = 8) were 297.0 ± 175.3 s up to 3 h. The mean duration of each SWD-like activity among animals exhibiting unilateral SWD-like activities was 34.9 ± 16.9 s. The number of each SWD-like activity between the animals was 8.3 ± 2.4 (Figure 1B).

### Mean duration of epileptiform activities and the sleep duration following dexmedetomidine injections

There was no significant difference in the length of epileptiform activity between “the unilateral SWD-like-exhibiting group” and “the non-SWD-like activities group” (Figure 1C). However, in the unilateral SWD-like-exhibiting group, mean sleep duration was considerably shorter than the non-SWD-like activities group (*t*_(df)_ = 3.812, *p* = 0.003, *p* < 0.01, Figure 1D).

### PSD analysis of SWD-like activity

**Figure 2:**
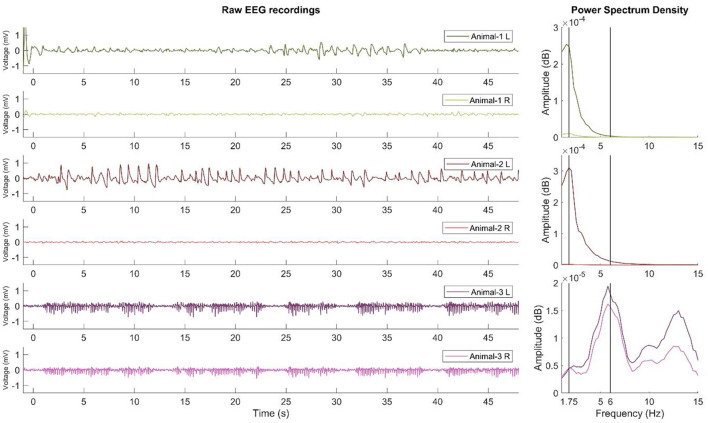
Raw recordings and PSD results of SWD and SWD-like activity. The left side of the figure shows the EEG recordings (filtered in 1–40 Hz) of two examples of SWD-like activity developed in the left hemisphere and one example of mature SWD activity recorded previously. Each subsequent two rows belong to the same animal. The right side of the figure shows the PSD results. As shown for the first two animals, SWD-like activity peaks at approximately 1.5–1.75 Hz, but for the third animal, the SWD activity peaks at 6 Hz and is similar on both hemispheres. L, left hemisphere; R, right hemisphere.

As shown in Figure 2, SWD-like activity is qualitatively similar to mature SWD activity, which classically indicates 6-Hz activity. However, SWD-like activity appears slower and peaks at 1.5–1.75 Hz. This activity developed only in the left hemispheres of 47% of the animals injected with the 0.6 mg/kg dexmedetomidine. Furthermore, the PSD results revealed that the 1.75 Hz power in the left hemisphere peaks significantly higher than that of the right hemisphere.

## Discussion

Generalized SWDs are known to be the basic building blocks of the EEG of absence epilepsy when manifested bilaterally and synchronously (25). Several genetic and pharmacological animal models have been constructed to understand the fundamental etiological factors behind epilepsies better and identify potential therapeutic targets for anti-epileptic medicines (26). GAERS, as an absence epilepsy model, provides a valuable model to examine the underlying epileptogenesis process in the pre-epileptic stage of the first 30 days of life in this animal model. This process is important for investigating potential anti-epileptogenic and anti-seizure treatment approaches and their use in creating new animal models.

Spike-and-wave discharges are high amplitude, synchronous, and, most significantly, bilateral. Previous studies were performed with unilateral cortical resection, but no change was reported. In contrast, SWDs were no longer noticeable after bilateral resection. These results suggest that SWDs are completely abolished after bilateral removal of the focal region, most likely by interfering with an intracortical columnar circuit (27), which also supports the cortical focus, whereas only inhibition of the local cortical network removed all seizures. Another unilateral onset is that SWDs have induced fluid percussion injury in rats, and it serves as a model for complex partial seizures in human post-traumatic epilepsy (28). In this model, anti-absence ethosuximide has been shown to suppress both unilateral or bilateral SWDs, whereas carbamazepine had no effect (28). Some drugs, such as potassium chloride, block SWDs somewhat in the ipsilateral cortex and thalamus (29). Furthermore, bilateral or unilateral SWDs have been observed to alternate between hemispheres after corpus callosum excision, implying that the corpus callosum is related to SWD generalization (30). Landau–Kleffner syndrome, commonly known as electrical status epilepticus of sleep, has focal SWDs (31). However, no report has shown unilateral induction of SWD-like activities with pharmacological or chemical agents.

In addition to the induction of unilateral seizures with dexmedetomidine, this study questions the focal origin hypothesis of absence seizures, specifically SWDs. Despite the absence of SWD activity observed in the baseline EEG of the pup GAERS, which is expected to be in their epileptogenesis period (before PN30), a dose of 0.6 mg/kg dexmedetomidine selectively induced unilateral SWD-like events in the left cortex of half of the animals. Animals that exhibited unilateral SWD-like activities accounted for 47% of the total sample. Unilateral expression of normally generalized seizures suggests a focal start. Some studies with dexmedetomidine also induced focal seizures suggest the activation of α_2A_AR may start the SWDs. In addition to these results, dexmedetomidine-inducing focal seizures in a periphery reflex model (20, 21) draw attention to α_2A_AR-mediated seizure initiation mechanisms.

Dexmedetomidine closely resembles and facilitates natural non-REM sleep (32) and therefore improves sleep in patients receiving dexmedetomidine anesthesia in comparison to other anesthetics after surgery by altering sleep structure (33, 34). Dexmedetomidine also modulates the release of inhibitory compounds such as γ-aminobutyric acid and galanin due to reduced control over the ventrolateral preoptic nucleus, further inhibiting the locus ceruleus and tuberomamillary nucleus by the inhibition and disinhibition of the locus ceruleus and ventral lateral preoptic nucleus (35). The α_2A_AR agonist effect of dexmedetomidine on NREM sleep might be influenced by postsynaptic α_2A_AR (36). Meanwhile, changes in high-frequency oscillations in the thalamus and neocortex have also been observed during dexmedetomidine anesthesia (37). In a genetic model of absence epilepsy, alterations in sleep characteristics were identified in WAG/Rij rats encompassing extended transitions from wakefulness to sleep, prolonged intermediate sleep stages, more frequent subsequent arousals, and a reduced proportion of REM sleep (38–40). That also points out the positive relationship between absence seizures and the increase of NREM activity as it is already known both rhythms of SWDs and NREM activities are synchronized in the thalamocortical circuitry (41, 42). The focal point pertains to the plausible role of dexmedetomidine in potentially inducing the transition of slow wave and delta oscillations, thereby precipitating SWDs.

A recent study on the mesoscale modeling of SWDs highlights and sheds light on the initiation, maintenance, and termination of SWDs by integrating pyramidal cells (43) and interneurons in the cortex (44, 45) as well as the ventroposterior medial nucleus of the thalamus, reticular thalamic nucleus (RTN), and nervus trigeminus (46). In this model, SWD might be initiated by one of the three mechanisms: an increase in intracortical excitability, external driving from the nervus trigeminus to the thalamic ventroposterior medial nucleus, or low-frequency harmonic stimulation of the cortex. While the maintenance was caused by increased coupling from RTN nodes to both pyramidal nodes and cortical interneurons (46), the termination was driven by increased coupling from rostral RTN to the brain or high-frequency electrical stimulation. We first demonstrated that SWD-like activity might be induced unilaterally using the α_2A_AR agonist dexmedetomidine. Though it appears to be the start of SWD-like activity in the left cortex, our previous study with a status epilepticus model found that dexmedetomidine generated sustained SWDs in adult rats, which is more consistent with the maintenance of SWD activity.

Our previous study (15) introduced this absence status epilepticus model with the induction of dexmedetomidine in adult GAERS. Recent reports on dexmedetomidine increasing the duration of SWD activity (15) provide evidence that dexmedetomidine influences SWD duration. For instance, Sitnikova et al. (47, 48) provided preliminary results of dexmedetomidine increasing mean duration of SWDs in another model of genetic absence epilepsy Wistar Albino Glaxo/Rijswijk (WAG/Rij), and dexmedetomidine does not shorten the SWDs unlike other anesthetics (49).

The mean sleep duration differed significantly between animals that exhibited unilateral SWD-like activities or not in this study as well. This difference can potentially be attributed to two distinct factors. First, anesthesia-induced sleep may interfere with the initiation of unilateral activities. Because our EEG recordings were obtained 2 h after injection, some animals may not fully emerge from the anesthesia during this timeframe. Second, given the metabolic differences among the animals, mainly as they are still in the pup stage, it is conceivable that the dosage of dexmedetomidine administered may have exceeded the specific activation threshold of α_2A_AR. These factors may have contributed to the observed difference in sleep duration between the animals.

Another issue on dexmedetomidine as addressed by many studies is the possible induction of respiratory or cardiovascular system-related adverse effects. Recent studies point out a either positive influence or no influence on the respiratory parameters (50–53). Conversely, a significant reduction in heart rate and instances of bradycardia have been reported as some of the cardiovascular effects (51). Yet, the relationship between them is yet to be investigated in terms of the sleep and SWD-related mechanisms.

As a result, it remains unclear whether the induction of unilateral SWD-like events reflects the initiation or maintenance phase of SWDs. In any case, α_2A_AR appears to be a strong candidate for further investigation as the primary mechanism behind the initiation or maintenance of SWDs as well as the modulation of switch mechanisms between sleep and SWDs. Further research into the exact cortical process and the role of α_2A_AR would be valuable.

## Data availability statement

The raw data supporting the conclusions of this article will be made available by the authors, without undue reservation.

## Ethics statement

The animal study was approved by Acibadem University Experimental Animals Local Ethics Committee under decision number ACUHAYDEK2020/51. The study was conducted in accordance with the local legislation and institutional requirements.

## Author contributions

MY and FO conceptualized and designed the study. MY organized the database, performed the statistical analysis, and wrote the first draft of the manuscript. SA performed the analysis and wrote the Results section of the PSD section of the manuscript. Pİ, NM, GD, ÖŞ, SK, and EK contributed to the EEG data acquisition. MY and FO contributed to the manuscript's revision and read and approved the submitted version. All authors approved the final version of the manuscript.
